# Coupling coordination evaluation and influencing factors analysis of older adult care service resource supply and demand in China

**DOI:** 10.3389/fpubh.2025.1662064

**Published:** 2025-11-26

**Authors:** Lingxiao Guo, Hua Wei, Qunshan Tao, Kunyu Chen

**Affiliations:** 1 School of Hospital Economics and Management, Anhui University of Chinese Medicine, Hefei, China; 2 Key Laboratory of Data Science and Innovative Development of Chinese Medicine in Anhui Province Philosophy and Social, Hefei, China

**Keywords:** older adult care service resources, supply and demand, coupling coordination, kernel density, influencing factors

## Abstract

**Background:**

The coupling coordination development between the supply and demand of older adult care service resources represents a critical pathway for the deep integration of the “Healthy China” initiative and the strategy of “actively responding to population aging,” and holds significant importance for achieving the modernization transformation of the older adult care service system. However, China’s current older adult care service system continues to face substantial challenges, including structural imbalances between supply and demand and uneven regional development. There is an urgent need to systematically evaluate the coupling coordination level between these two systems and identify key influencing factors to provide theoretical support for evidence-based decision-making.

**Methods:**

Based on panel data from 31 provinces and municipalities in China spanning 2014–2023, this study employs the entropy weight method to construct a comprehensive evaluation index system for the supply and demand of older adult care service resources. The coupling coordination degree model is utilized to measure the coordinated development level between the two systems, and a Tobit panel model is applied to identify key factors influencing coupling coordination development.

**Results:**

The coupling coordination degree between the supply and demand of older adult care service resources in China exhibits an overall upward trend, having advanced from the verge of imbalance to barely coordinated status; however, the overall level remains relatively low. The spatial pattern demonstrates a significant “high in the east, low in the west” distribution characteristic, with the eastern region having reached primary coordination level, while the central and western regions predominantly remain in a supply-lagging imbalanced state. Economic development level emerges as the primary driving factor affecting coupling coordination, with science and technology expenditure, social capital investment, and education level all playing significantly positive roles, while the efficiency of government fiscal expenditure requires improvement.

**Conclusion:**

To enhance overall coordination levels and achieve balanced regional development, policymakers should formulate differentiated development strategies based on local resource endowments, optimize the collaborative mechanism between supply and demand of older adult care service resources, improve cross-departmental collaborative governance systems, and promote high-quality development of the older adult care service system.

## 1 Introduction

The scientific, efficient, and rational allocation of older adult care service resources represents a critical issue for addressing the challenges of population aging, ensuring quality of life for older adults, promoting social harmony and stability, and advancing sustainable national socioeconomic development (1). According to data from the National Bureau of Statistics, by the end of 2023, China’s population aged 60 and above had reached 297 million, accounting for 21.1% of the total population, indicating that China had entered a stage of advanced aging. However, China’s current older adult care service system continues to face numerous challenges, including irrational supply structures, inconsistent service quality, imbalanced urban–rural development, and insufficient integration of medical and nursing care services. To better address these challenges, in 2022, the State Council explicitly articulated in the “14th Five-Year Plan for the Development of Aging Services and Older adult care service System” the objective to “establish an older adult care service system that coordinates home-based, community-based, and institution-based care while integrating medical and nursing care services” to achieve dynamic equilibrium between the supply and demand of older adult care services and meet the multi-level and diversified needs of older adults (2). Therefore, promoting the coordinated development of older adult care service resource supply and demand and providing high-quality, convenient, and affordable older adult care services has become a pressing issue requiring urgent attention (3).

Existing research demonstrates that the supply of older adult care service resources significantly promotes demand development. At the infrastructure level, Wei LJ et al. found that increases in the number of older adult care institutions and facilities not only directly expanded service supply capacity but also reduced service costs and enhanced service accessibility through economies of scale (4). Yu C research indicates that the development of integrated medical-nursing services has effectively consolidated medical and older adult care resources, providing more specialized care support for disabled older adult (5). Using spatial econometric models, Luo D et al. discovered that rational facility layout could significantly enhance life satisfaction among older adults while generating pronounced spatial spillover effects (6). The empirical study by Zhang YA et al. revealed that the application of smart older adult care platforms, intelligent monitoring devices, and remote health management systems has played important roles in improving service efficiency and optimizing resource allocation (7). Through cross-national comparative research, Korfhage T et al. demonstrated that government fiscal investment strengthened the public welfare and inclusiveness of older adult care services by enhancing the social security system (8).

International comparative studies have further enriched understanding of supply-side mechanisms. Saito T et al.’s empirical research on Japan’s long-term care insurance system demonstrated that institutionalized supply capacity building could effectively respond to dynamic demand changes (9). Studies examining Germany’s tiered care system revealed that differentiated supply strategies could significantly improve resource allocation efficiency (10). Gammon D et al.’s research emphasized that Norway’s community service network played a crucial role in enhancing older adult care service accessibility (11). Jain M et al.’s examination of Singapore’s reforms revealed that innovative supply models could reshape demand structures and promote service utilization (12). However, existing research primarily focuses on the unidirectional effect of supply on demand, treating demand as a passive recipient, while lacking systematic theoretical elucidation of the interactive feedback mechanisms between supply and demand. This research gap constrains our in-depth understanding of the dynamic evolutionary patterns of older adult care service systems.

Although existing research has thoroughly explored supply-side effects, theoretical research on how the demand side reciprocally shapes the supply system remains markedly insufficient. In practice, the intensification of population aging and expansion of the older adult population directly drive growth in older adult care service market demand, providing market impetus for supply-side development; the diversification of older adults’ demand structures and enhancement of consumption capacity provide direction for supply quality upgrading; the rising proportion of disabled older adults and increase in empty-nest families generate specialized service demands, reciprocally promoting improvements to the supply system (13). This study constructs a theoretical framework for the bidirectional coupling of older adult care service resource supply and demand, systematically elucidating the interactive mechanisms and coordination patterns between the two systems, which holds important theoretical value and global significance. At the theoretical level, this research transcends the traditional unidirectional analysis paradigm, revealing the dynamic evolution mechanism of bidirectional supply–demand interaction from a systems theory perspective, expanding the application boundaries of coupling coordination theory in the social service field, and providing a universal analytical tool for comparative research on older adult care service systems under different national contexts. At the global aging governance level, based on empirical analysis of China’s ultra-large-scale population, this research reveals the path mechanism for achieving supply–demand coordination under resource constraints in developing countries, offering a referenceable theoretical framework and policy insights for countries facing similar challenges. At the empirical level, based on provincial panel data, this research identifies the spatiotemporal evolution patterns and driving factors of supply–demand coupling coordination, providing evidence-based support for formulating differentiated regional policies, with its methodology holding reference value for other transition economies. Therefore, this research not only deepens theoretical understanding of older adult care service supply–demand relationships but also provides systematic solutions for service system optimization in the context of global aging.

## 2 Coupling coordination mechanism of older adult care service resource supply and demand in China

**Figure 1:**
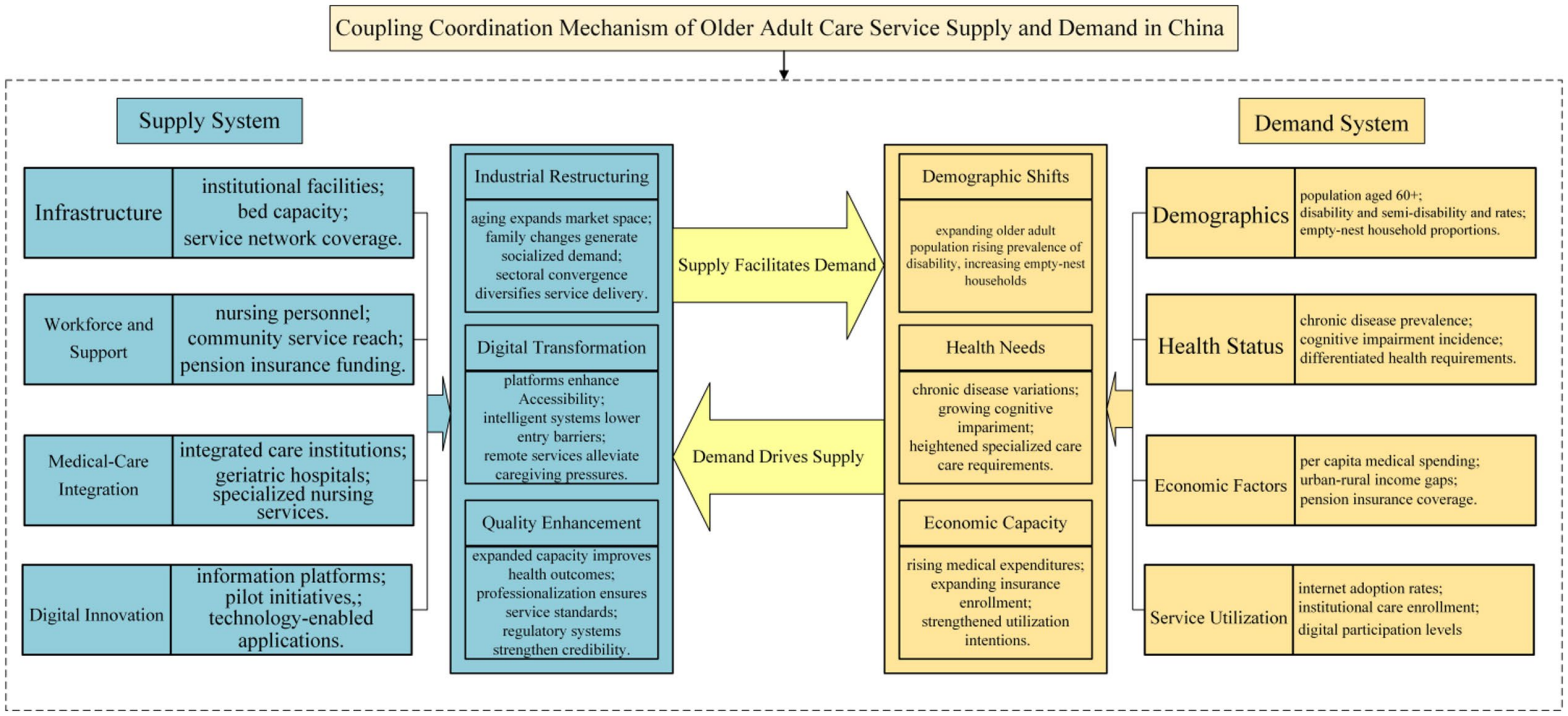
Coupling coordination theoretical model.

Based on systems theory and synergetics, the supply and demand of older adult care service resources constitute an interdependent and dynamically evolving complex system. Existing research has predominantly focused on unidirectional effects; this study constructs a bidirectional coupling framework to elucidate the intrinsic mechanisms through which the two systems achieve synergistic evolution via resource allocation, information feedback, and policy regulation (Figure 1).

### 2.1 Older adult care service resource supply effectively promotes demand development

As an essential prerequisite for demand release and development, older adult care service resource supply plays a crucial role in promoting the formation and expansion of older adult care service demand through industrial structural optimization, technological innovation application, and quality assurance system construction. From the perspective of supply-side structural reform, the promotion mechanism of older adult care service resource supply on demand development is primarily reflected in the following three dimensions:

(1) Industrial structural transformation creates development opportunities for older adult care service demand
  The structural adjustment and upgrading of the older adult care service industry provide important external conditions and internal impetus for demand release (14). The accelerated advancement of population aging has directly expanded the potential demand scale for older adult care services, creating enormous market space for industrial development. Profound changes in family structure, particularly the prevalence of nuclear family structures and the rising proportion of empty-nest older adult households, have weakened traditional family care functions, thereby generating rigid demand for socialized and specialized older adult care services (15). The sustained increase in income levels has strengthened the payment capacity of the older adult population, establishing an economic foundation for the release of mid-to-high-end older adult care service demand. The deep integration of older adult care services with healthcare, smart technology, cultural education, and wellness tourism industries has spawned emerging formats such as integrated medical-nursing care, smart older adult care, and wellness tourism, enriching both the supply forms and content levels of older adult care services. The increase in the number of integrated medical-nursing institutions and the construction of geriatric hospitals directly respond to the specialized nursing care demands generated by the rising proportion of disabled/semi-disabled older adult populations. The expansion in the number of universities for older adults not only satisfies the spiritual and cultural needs of older adults but also drives the emergence of older adult education industry, further expanding effective demand for older adult care services.
(2) Digital transformation and upgrading lead new models of older adult care service demand.
  Digital transformation plays an increasingly important role in promoting supply–demand matching in older adult care services. The improvement in older adult care information platform coverage and the increase in the number of smart older adult care pilot cities have not only enhanced the convenience and precision of older adult care services but have also stimulated new service demands through technological empowerment (16). The application of smart older adult care technology has lowered the threshold for older adults to access services. Particularly for empty-nest older adult households, intelligent services such as remote monitoring, health management, and emergency response have effectively alleviated family care pressures while enhancing the capacity and confidence of older adults to live independently. The innovation of digital service models has also increased internet usage rates among the older adult population, advancing the degree of digital participation among older adult groups. Through digital means such as online appointment booking, remote consultation, and intelligent assessment, older adults can more conveniently access personalized and specialized older adult care services. This convenience, in turn, stimulates growth in demand for high-quality older adult care services (17).
(3) Service quality improvement and regulatory system enhancement provide solid guarantees for demand development.
  The continuous improvement of older adult care service quality constitutes a core element in stimulating and sustaining demand growth (18). According to human capital theory, health, as an important stock of human capital, retains significant economic and social value in later life. The increase in the number of beds per thousand older adults and the expansion in the number of integrated medical-nursing institutions directly enhance the health management level and medical care quality for older adults, effectively extending both the health lifespan and independent living capacity of the older adult population. The improvement in community older adult care service institution coverage enables older adults to access professional health management and life care services locally, significantly enhancing the overall health status and quality of life of the older adult population. The increase in the certification rate of older adult care workers promotes the development of older adult care service toward specialization and standardization, effectively ensuring the stability and reliability of service quality. A comprehensive quality supervision system not only strengthens the trust of older adults and their families in older adult care services but also provides institutional guarantees for the sustained supply of high-quality older adult care services by establishing service standards, standardizing operational procedures, and strengthening accountability mechanisms. This quality assurance mechanism has promoted steady growth in the total number of older adults residents in institutions at year-end, reflecting improved acceptance of and satisfaction with institutionalized older adult care services among older adults.

### 2.2 Older adult care service demand serves as the fundamental driver of resource supply development

Against the backdrop of population aging, older adult care service demand exerts a decisive constraining and guiding effect on the development of the supply system. The scale, structure, and levels of older adult care service demand directly influence the investment direction, resource allocation, and service model innovation on the supply side. The driving effect of demand on supply is primarily evident in the following three aspects:

(1) Demographic structural changes drive upgrading and optimization of older adult care service supply structure.
  The rapid growth in the population aged 60 and above and the rising proportion of disabled and semi-disabled older adult populations directly drive the expansion of total older adult care service supply and structural adjustment (19). Generally, regions with larger older adult populations demonstrate greater investment in the number of older adult care institutions and facilities. As the degree of population aging deepens, governments increase fiscal investment in older adult care services, and social capital increasingly enters the older adult care service market, promoting increases in the number of older adult care institutions and service capacity. The rising proportion of empty-nest older adult households has exerted profound impacts on supply structure, driving the rapid development and improvement in coverage of community older adult care service institutions (20). To meet the specialized nursing care needs of disabled and semi-disabled older adults professional service facilities such as integrated medical-nursing institutions and geriatric hospitals have developed rapidly, promoting the upgrading of older adult care service supply toward specialization and medicalization while injecting new vitality into the older adult care service industry.
(2) Differentiated health needs drive precise allocation of medical-nursing service resources.
  Regional differences in chronic disease prevalence rates and standardized incidence rates of Alzheimer’s disease among the older adult population directly influence the supply layout and resource allocation strategies of integrated medical-nursing services (21). Regions with higher chronic disease prevalence rates face more urgent demands for long-term care services, driving the construction of integrated medical-nursing institutions and the cultivation of professional nursing personnel (22). The rising incidence rates of Alzheimer’s disease and other cognitive impairment diseases have generated demands for specialized memory care services, promoting the development of relevant specialized institutions and service programs. These differentiated characteristics of health needs require that older adult care service supply possess stronger specialization and precision capabilities, driving improvements in the certification rate of older adult care workers and professional skill enhancement while promoting the innovative development of integrated medical-nursing service models (23).
(3) Consumption capacity and payment willingness determine the quality levels of older adult care service supply.
  The growth in per capita medical consumption expenditure levels and the number of participants in urban basic pension insurance at year-end among the older adult population directly influences the market positioning and service levels of older adult care service supply (24). Older adults with better economic conditions directly select high-quality older adult care services, driving the development of the high-end older adult care service market and the improvement of service standards. Changes in the urban–rural older adult income disparity coefficient affect the balanced development of urban–rural older adult care service supply; regions with larger income disparities require the establishment of more diversified service supply systems. The total number of older adult residents in institutions at year-end, as an important indicator of service utilization, reflects the actual demand for and acceptance of institutionalized older adult care services among older adults. Changes in this indicator directly influence the construction scale, bed allocation, and service capacity building of older adult care institutions, driving the supply side to adjust development strategies and resource allocation priorities based on actual utilization patterns.

### 2.3 Bidirectional supply–demand interaction promotes coupled and coordinated development

Overall, the supply and demand of older adult care service resources do not constitute a unidirectional promotion relationship but rather exhibit close bidirectional interactive effects (25). The supply system provides a solid material foundation and technical support for demand release and development through infrastructure construction, personnel and security system improvement, integrated medical-nursing service development, and smart older adult care technology application. The demand system provides directional guidance and market impetus for the optimization and upgrading of the supply system through demographic structural changes, health status differences, consumption capacity enhancement, and service utilization feedback. From the perspective of system dynamics, bidirectional supply–demand interaction forms a positive feedback loop: improved supply capacity promotes effective demand release; demand release expands market scale; market scale expansion attracts more resource investment to the supply side, thereby further enhancing supply capacity. From the perspective of synergetics, the two systems achieve order parameter synergy through information flow, capital flow, and service flow. When the degree of synergy exceeds a critical threshold, the system enters a high-level coupling state, manifested as a benign development trajectory characterized by matched supply–demand structures, efficient resource allocation, and excellent service quality. This theoretical framework transcends traditional linear supply–demand analysis, providing a systematic explanatory tool for understanding the dynamic evolution of China’s older adult care service system.

## 3 Data and methods

### 3.1 Indicator selection

#### 3.1.1 Selection of indicators for older adult care service resource supply in China

This study draws upon comprehensive evaluation indicator selection from previous related research. Based on a synthesis of relevant research findings and according to indicator availability, independence, and scientific rigor, this study establishes a coupling coordination evaluation indicator system. The system encompasses two major dimensions—older adult care service resource supply and demand—and aims to comprehensively reflect the development levels of both systems and their degree of coordination.

Infrastructure serves as the material foundation of older adult care service supply, directly influencing the supply capacity and coverage of older adult care services (26). The number of older adult care institutions and facilities reflects the basic supply scale of older adult care services. The number of beds per thousand older adults represents the per capita allocation level of older adult care service resources and serves as a core indicator for measuring the adequacy of older adult care service supply. The number of universities for older adults not only reflects the supply level of spiritual and cultural older adult care services but also demonstrates the practical implementation of the active aging concept. These three indicators represent the accessibility boundaries of the service system, resource allocation efficiency, and the developmental transformation of service philosophy, respectively.

Personnel and security levels serve as important support for ensuring older adult care service quality (27). The certification rate of older adult care workers directly reflects the professionalization level and service quality assurance capacity of older adult care services. The coverage rate of community older adult care service institutions reflects the accessibility and convenience of home-based and community-based older adult care services, holding a pivotal position in China’s home-centered older adult care framework. Basic pension insurance expenditure, as an important financial guarantee for older adult care services, reflects the support capacity of the social security system for older adult care service supply, serving as a key link connecting supply capacity building and demand purchasing power (28).

The development level of integrated medical-nursing care represents an important characteristic of modern older adult care service systems (29). The number of integrated medical-nursing institutions reflects the degree of integrated development of medical and older adult care services. The number of geriatric hospitals reflects the supply capacity of specialized geriatric medical services. These indicators collectively measure the responsiveness of older adult care service supply to the health needs of older adults (30). As the proportion of disabled and semi-disabled older adult populations continues to rise, the deep integration of medical care and older adult care services has become a structural requirement that the supply side must address.

The construction level of smart older adult care represents the modernization development direction of older adult care services (31). The coverage rate of older adult care information platforms reflects the degree of popularization of information technology application in older adult care services. The number of smart older adult care pilot cities reflects the innovative practice level of smart older adult care models. These indicators can measure the technological content and developmental foresight of older adult care service supply, constituting key dimensions for evaluating the progress of supply-side digital transformation (32).

#### 3.1.2 Selection of indicators for older adult care service resource demand in China

Demographic structural characteristics constitute the fundamental determinants of older adult care service demand (33). The population aged 60 and above directly reflects the potential demand scale for older adult care services. The proportion of disabled/semi-disabled older adult populations reflects the intensity of demand for specialized nursing care services. The proportion of empty-nest older adult households reflects the urgency of socialized older adult care services. These indicators collectively constitute the demographic foundation of older adult care service demand.

Differentiated health needs represent important characteristics of older adult care service demand (34). The chronic disease prevalence rate among the older adult population reflects the basic demand level for medical and nursing care services. The standardized incidence rate of Alzheimer’s disease reflects the specialized demand for professional cognitive care services (35). These health indicators accurately reflect the degree of demand for differentiated and specialized older adult care services among the older adult population (36).

Economic and consumption capacity serve as key factors for transforming older adult care service demand into effective demand (37). Per capita medical consumption expenditure among the older adult population reflects the actual consumption capacity and payment willingness of the older adult population. The urban–rural older adult income disparity coefficient reflects the spatial distribution characteristics of demand. The number of participants in urban basic pension insurance at year-end reflects the supporting role of institutionalized security in demand release.

The level of digital participation and service utilization reflects the modernization characteristics of older adult care service demand (38). The internet usage rate among the older adult population reflects the capacity foundation of the older adult population for accessing and utilizing digital older adult care services (39). Internet usage capacity directly influences the extent to which older adults realize demands for digital services such as telemedicine, online consultation, and intelligent monitoring. Regional differences in usage rates essentially reflect the capacity differentiation among different older adult groups in service access opportunities (40). Meanwhile, internet usage behavior is closely related to the health literacy, information acquisition capacity, and service-seeking willingness of older adults, comprehensively reflecting the acceptance and adaptability of older adults groups to innovative service models such as smart older adult care and integrated medical-nursing care (41). This indicator not only measures the current realistic demand scale for digital services but also reflects the potential space for future transformation of older adult care service demand toward intelligence and convenience. The total number of older adult residents in institutions at year-end directly reflects the actual utilization of institutionalized older adult care services, constituting an objective measure of explicit demand. Together with internet usage rates, these two indicators form a complementary measurement system of potential demand capacity and realized demand (Table 1).

**Table 1:** Coupling coordination evaluation indicator system for older adult care service resource supply and demand in China.

Target system	Primary indicator	Secondary indicator (Unit)	Weight	Direction
Supply	Infrastructure	Number of older adult care institutions and facilities (units)	0.238	+
		Number of beds per thousand older adults (beds)	0.047	+
		Number of universities for the older adults (institutions)	0.031	+
	Personnel and Security	Certification rate of older adult care workers (%)	0.051	+
		Coverage rate of community older adult care service institutions (%)	0.119	+
		Basic pension insurance expenditure (hundred million yuan)	0.144	+
	Medical-Nursing Integration	Number of integrated medical-nursing institutions (institutions)	0.083	+
		Number of geriatric hospitals (institutions)	0.155	+
	Smart Older Adults Care	Coverage rate of older adult care information platforms (%)	0.044	+
		Number of smart older adult care pilot cities (cities)	0.088	+
Demand	Demographic Structure	Population aged 60 and above (10,000 persons)	0.181	+
		Proportion of disabled/semi-disabled older adult population (%)	0.062	+
		Proportion of empty-nest older adult households (%)	0.060	+
	Health Status	Chronic disease prevalence rate among the older adults (%)	0.038	+
		Standardized incidence rate of Alzheimer’s disease (%)	0.105	+
	Economy and Consumption	Per capita medical consumption expenditure among the older adults (yuan)	0.077	+
		Urban–rural older adult income disparity coefficient	0.047	−
		Number of participants in urban basic pension insurance at year-end (10,000 persons)	0.061	+
	Digital Participation	Internet usage rate among the older adults (%)	0.175	+
	Service Utilization	Total number of older adult residents in institutions at year-end (persons)	0.195	+

#### 3.1.3 Influencing factors of coupling coordination between older adult care service resource supply and demand in China

Based on existing research and data availability, as well as the “Several Measures to Promote High-Quality Development of Inclusive Older Adult Care Services” and the “Opinions on Promoting the Construction of Basic Older Adult Care Service Systems,” this study selects key factors that may influence the coupling coordination development of older adult care service supply and demand in China, including economic development level, government support intensity, and sociodemographic structure. Ultimately, per capita GDP, government fiscal expenditure on older adult care services, older adults dependency ratio, proportion of social capital investment in older adult care industry, fiscal education expenditure, and per capita disposable income of older adults were selected as specific analytical variables (Table 2).

**Table 2:** Influencing factors of coupling coordination development between supply and demand of older adult care service resources in China.

Variable category	Variable name	Variable symbol	Unit	Variable description
Explained variable	Coupling coordination degree	D	–	Calculated results of coupling coordination degree between the two systems
Explanatory variables	Economic factor	PGD	yuan/person	Per capita GDP
	Policy regulation	GES	10,000 yuan	Government fiscal expenditure on older adult care services
	Social structure	ODR	%	Older adults dependency ratio
	Social support	SCR	%	Proportion of social capital investment in the older adults care industry
	Education expenditure	FEE	Hundred million yuan	Fiscal education expenditure
	Consumption capacity	SDI	10,000 yuan	Per capita disposable income of the older adults

### 3.2 Research methods

#### 3.2.1 Entropy weight method

The entropy weight method is an objective weighting method based on the degree of data dispersion, capable of calculating the comprehensive development scores of older adult care service resource supply and demand in China (42). The specific calculation steps include normalizing each indicator to ensure comparability among different indicators; then calculating the entropy value of each indicator to reflect the redundancy of its information entropy (i.e., the variability among indicators); finally, calculating the weight of each indicator based on the entropy value to obtain the comprehensive scores of the supply side (U₁) and demand side (U₂) (43). This method avoids interference from subjective human factors in weight allocation, enhancing the scientific rigor and objectivity of the evaluation process. The specific calculation procedures are as follows:

(1) Data normalization:

When Xij is a positive indicator (Equation 1):


(1) $$X_{\mathrm{ij}}=\frac{X_{\mathrm{ij}}-\min(X_{\mathrm{ij}})}{\max(X_{\mathrm{ij}})-\min(X_{\mathrm{ij}})}$$

When Xij is a negative indicator (Equation 2):


(2) $$X_{\mathrm{ij}}=\frac{\max(X_{\mathrm{ij}})-X_{\mathrm{ij}}}{\max(X_{\mathrm{ij}})-\min(X_{\mathrm{ij}})}$$

(2) Weight calculation (Equation 3):

(3) $$\begin{array}{l} P_{\mathrm{ij}}=\frac{X_{\mathrm{ij}}}{\sum_{i=1}^{n}X_{\mathrm{ij}}} \end{array}$$

(3) Entropy value calculation (Equation 4):

(4) $$\begin{array}{l} E_{j}=-\frac{1}{\mathrm{lnn}}\sum_{i=1}^{n}P_{\mathrm{ij}}\ln(P_{\mathrm{ij}}) \end{array}$$

(4) Weight assignment (Equations 5, 6):

(5) $$d_{j}=1-E_{j}$$

(6) $$\begin{array}{l} W_{j}=\frac{d_{j}}{\sum_{j=1}^{m}d_{j}} \end{array}$$

(5) Comprehensive score calculation (Equation 7):

(7) $$U=\sum_{i=1}^{m}W_{j}X_{\mathrm{ij}}$$

In the formulas, $X_{\mathrm{ij}}$ represents the observed value of the jth indicator in the ith province or municipality; m represents the number of indicators used; $W_{j}$ represents the weight of the jth indicator; U represents the comprehensive development level score for supply and demand; $U_{1}$ represents the comprehensive score for supply; and $U_{2}$ represents the comprehensive score for demand.

#### 3.2.2 Relative development degree

Based on the standardized data and weight allocation results obtained from the entropy weight method, this study constructs a comprehensive development level evaluation system for the two major systems of older adult care service resource supply and demand in China (44). By calculating the supply system comprehensive development level index U₁ and the demand system comprehensive development level index U₂, and using the ratio r of the two as a relative development degree indicator, this study systematically identifies the coordination status and structural characteristics of older adult care service resource supply–demand development across regions.

Based on the differential manifestations of relative development degree value intervals, the developmental trends of older adult care service resource supply and demand across provinces and municipalities nationwide are categorized into the following three typical patterns: Specifically, if 0 < r ≤ 0.8, it indicates that older adult care service resource supply is relatively lagging, belonging to the supply-lagging type; when 0.8 < r ≤ 1, it indicates that supply and demand are in a state of synchronized development, belonging to the synchronized development type; whereas when r > 1, it indicates that the demand for older adult care service resources is relatively lagging, belonging to the demand-lagging type. This ratio provides an important reference basis for evaluating the relative development levels of the two subsystems (Equation 8).


(8) $$\begin{array}{l} r=\frac{U_{1}}{U_{2}} \end{array}$$

#### 3.2.3 Coupling coordination degree model

The coupling coordination degree model is a quantitative analysis method used to evaluate the interaction intensity and coordinated development level between systems. Based on the concept of “coupling” in physics, this model measures the degree of synergy and development quality between different systems through mathematical modeling (Equations 9–11). This study applies this model to analyze the coupling coordination relationship between the two subsystems of older adult care service resource supply and demand in China (45). The specific calculation procedures are as follows:


(9) $$C=2\sqrt{\frac{U_{1}\times U_{2}}{{\left(U_{1}+U_{2}\right)}^{2}}}$$

(10) $$D=\sqrt{C\times T}$$

(11) $$T=\alpha U_{1}+\beta U_{2}$$

In China’s older adult care service system, the supply system and demand system possess equal importance and influence; therefore, this study sets α=β=0.5 , ensuring the standardization constraint of α+β=1 . The values of coupling degree C and coupling coordination degree D are both distributed within the [0,1] interval, where $U_{1}$ and U₂, as the comprehensive evaluation scores of the two subsystems, are likewise constrained to the [0,1] range. Higher values of C, D, $U_{1}$ , and $U_{2}$ indicate higher levels of coordinated development between the two systems. Based on the classification standards of relevant research and combined with the development characteristics of China’s older adult care services, this study categorizes coupling coordination degree into different levels and development stages (Table 3).

**Table 3:** Classification standards for coupling coordination degree between older adult care service resource supply and demand in China.

Coupling coordination degree D	Coupling coordination level	Interval type
[0.00–0.10)	Extreme imbalance	Low-level coupling stage
[0.10–0.20)	Severe imbalance	
[0.20–0.30)	Moderate imbalance	
[0.30–0.40)	Mild imbalance	Antagonistic stage
[0.40–0.50)	Near imbalance	
[0.50–0.60)	Marginal coordination	Running-in stage
[0.60–0.70)	Preliminary coordination	
[0.70–0.80)	Intermediate coordination	
[0.80–0.90)	Good coordination	High-level coupling stage
[0.90–1.00]	Excellent coordination	

#### 3.2.4 Kernel density estimation

Kernel density estimation is a non-parametric testing method commonly used for the intuitive visualization of continuous data and represents an ideal method for measuring the evolution of coupling coordination degree between older adult care service resource supply and demand (46). Therefore, this study employs kernel density curves to depict the distribution position, morphology, and extensibility of the coupling coordination degree between older adult care service resource supply and demand. Its basic formula is (Equation 12):


(12) $$f(x)=\frac{1}{\mathrm{Nh}}\sum_{j=1}^{N}k(\frac{x_{j}-x}{h})$$

where N is the sample size, h is the bandwidth, and x is the mean. This study employs MATLAB software to generate kernel density plots, with bandwidth settings as follows: national 0.03, eastern 0.04, central 0.03, western 0.03. The distribution position indicates the level of coupling coordination between older adult care service resource supply and demand in a region; the number of peaks indicates the degree of polarization in regional older adult care service resource supply–demand coupling coordination; peak height indicates the magnitude of disparity in regional older adult care service resource supply–demand coupling coordination levels; and distribution extensibility indicates the gap between the highest and lowest level individuals in regional older adult care service resource supply–demand coupling coordination.

#### 3.2.5 Tobit panel model with provincial and time fixed effects

Since the value range of the coupling coordination degree falls between 0 and 1, it constitutes a limited dependent variable (47). Therefore, this study employs a provincial and time two-way fixed-effects Tobit panel model to analyze the influencing factors of the coupling coordination degree between older adult care service resource supply and demand in China. This model simultaneously controls for provincial fixed effects and time fixed effects to mitigate omitted variable bias and temporal trend interference. To avoid multicollinearity and heteroscedasticity issues, this study applies logarithmic transformation to all explanatory variables. The calculation formula is as follows:


(13) $$Y_{\mathrm{it}}=\alpha +\sum_{j=1}^{m}\beta_{j}\ln X_{\mathrm{jit}}+\mu_{i}+\lambda_{t}+\varepsilon_{\mathrm{it}}$$

where $Y_{\mathrm{it}}$ represents the coupling coordination degree; α is the constant term; $\beta_{j}$ represents the coefficient of each explanatory variable; $\ln X_{\mathrm{jit}}$ represents the explanatory variables; $\mu_{i}$ represents provincial fixed effects; $\lambda_{t}$ represents time fixed effects; i and t represent province and time, respectively; and $\varepsilon_{\mathrm{it}}$ is the random disturbance term.

Additionally, to verify the robustness of the results, this study employs a two-way fixed-effects Tobit model with one-period lagged explanatory variables for robustness testing, with results provided in the Supplementary materials.

### 3.3 Data sources

Based on data comprehensiveness and availability, this study selects data from 31 provinces, autonomous regions, and municipalities in China from 2014 to 2023 as research samples. Relevant older adult care service resource data are sourced from the China Statistical Yearbook (2015–2024), China Civil Affairs Statistical Yearbook, China Human Resources and Social Security Yearbook, China Health and Hygiene Statistical Yearbook, China Social Insurance Yearbook, China Social Service Statistical Yearbook, White Paper on Smart Health and Older Adult Care Industry Development, Statistical Bulletin on the Development of Aging Services in China, and Statistical Report on Internet Development in China. According to the classification standards of the China Statistical Yearbook, China is divided into three major regions: eastern, central, and western.

## 4 Research results and analysis

### 4.1 Analysis of the comprehensive development levels of the two systems of older adult care service resource supply and demand in China

**Figure 2:**
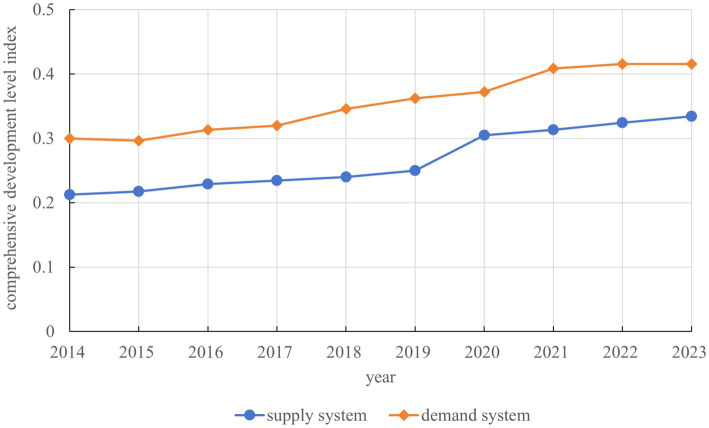
Comprehensive development levels of older adult care service resource supply and demand in China.

From the perspective of overall development trends, during the period 2014–2023, the comprehensive development levels of both the supply and demand systems of older adult care service resources in China exhibited sustained upward trends, though the development trajectories of the two systems demonstrated differentiated characteristics. The development level of the demand system consistently led that of the supply system, and this “demand-driven” development model reflected the strong demand-pull effect of the population aging process on older adult care services. However, the supply system demonstrated a faster growth rate, and the converging trend in the development gap between the two systems indicated continuously strengthening supply–demand coordination, with the accelerated development since 2019, in particular, injecting new momentum into this coordination process (Figure 2).

**Figure 3:**
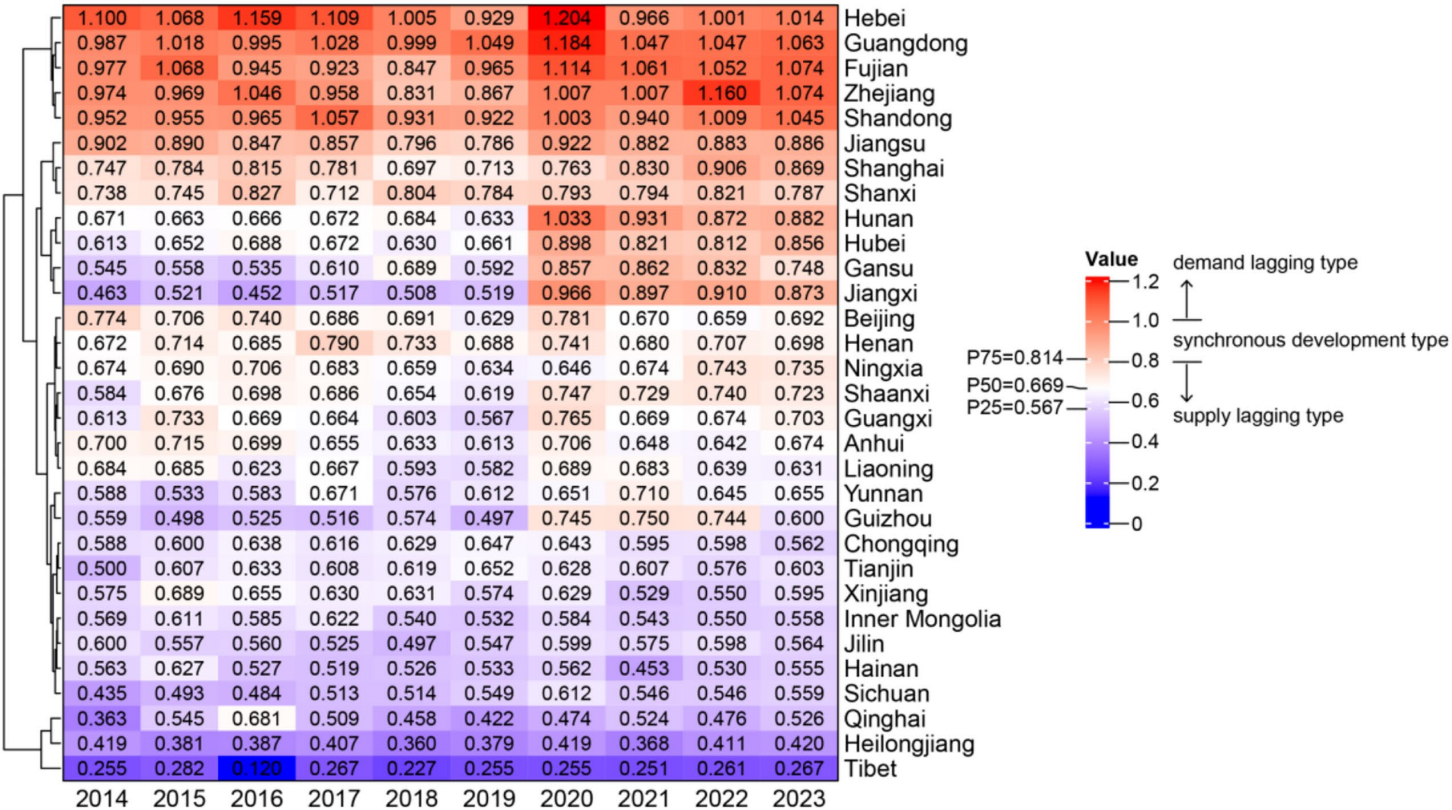
Relative development degree of older adult care service resource supply and demand in China.

Based on the spatiotemporal evolution characteristics revealed by the heat map analysis (Figure 3), the relative development degree of older adult care service resource supply and demand in China from 2014 to 2023 exhibited pronounced spatiotemporal differentiation patterns and staged evolutionary characteristics.

From the temporal dimension, the relative development degree of older adult care service resource supply and demand underwent distinct fluctuating evolution. From 2014 to 2018, the heat map displayed an overall pattern dominated by blue-green tones, with r values for most provinces nationwide concentrated in the 0.5–0.9 range. Among them, provinces such as Hebei and Guangdong had already reached values above 1.0, classified as the demand-lagging type, while western provinces such as Tibet and Qinghai had r values below 0.4, belonging to the severely supply-lagging type. The year 2019 became a critical juncture in supply–demand relationship evolution, with r values declining in most provinces and heat map colors shifting toward blue, indicating a dynamic adjustment of supply–demand relationships. From 2020 to 2023, the relative development degree of supply and demand exhibited a differentiated pattern: r values in eastern provinces gradually recovered and stabilized in the 0.8–1.1 range, displaying orange-red tones; central provinces maintained values in the 0.6–0.8 range, showing yellow-green tones; while western provinces remained persistently at low levels in the 0.3–0.6 range, with the heat map displaying deep blue tones, and regional disparities continued to expand.

In terms of spatial distribution, the relative development degree of older adult care service resource supply and demand exhibited a geographical pattern of “eastern high, western low, with three-tier gradients.” The eastern region demonstrated the most outstanding performance, with provinces such as Shandong, Guangdong, and Hebei maintaining relatively high r values throughout the study period. In 2023, the r values for Shandong, Guangdong, and Hebei reached 1.095, 1.063, and 1.014, respectively, all classified as the demand-lagging type, with the heat map displaying stable red high-value zones. The central region exhibited transitional characteristics, with provinces such as Henan, Hubei, and Hunan showing r values fluctuating in the 0.6–0.9 range. In 2023, these values stood at 0.898, 0.862, and 0.787, respectively, with the heat map displaying yellow-green tones, reflecting relatively balanced supply–demand relationships in this region. The western region demonstrated relatively lagging development levels with pronounced internal differentiation. Sichuan and Chongqing had relatively higher r values (0.559 and 0.562, respectively, in 2023), while Tibet’s r value remained persistently at extremely low levels below 0.3 (0.267 in 2023), and Qinghai’s r value hovered around 0.4 (0.420 in 2023). The heat map displayed deep blue low-value zones, indicating that factors such as geographical environment and economic development levels imposed significant constraints on supply capacity building.

### 4.2 Coupling coordination degree between older adult care service resource supply and demand in China

The coupling coordination degree results for older adult care service resource supply and demand across provinces and municipalities in China are presented in Table 4. From the national perspective, the coordination degree between older adult care service resource supply and demand in China from 2014 to 2023 exhibited a clear upward trend. Examining regional variations, the eastern region overall demonstrated a relatively stable upward trajectory; the central region experienced minor fluctuations in the early period before entering a stage of steady growth in the later period; while the western region exhibited a development trajectory characterized by gradual initial increases followed by subsequent plateauing.

**Table 4:** Coupling coordination degree and coordination types between older adult care service resource supply and demand in China, 2014–2023.

Region	Province	Year										Coordination type	
		2014	2015	2016	2017	2018	2019	2020	2021	2022	2023	2014	2023
Eastern	Beijing	0.559	0.565	0.575	0.583	0.607	0.603	0.606	0.621	0.621	0.639	Marginal coordination	Preliminary coordination
	Tianjin	0.464	0.465	0.486	0.493	0.506	0.506	0.519	0.527	0.538	0.545	Near imbalance	Marginal coordination
	Hebei	0.556	0.573	0.597	0.595	0.625	0.635	0.671	0.706	0.707	0.722	Marginal coordination	Intermediate coordination
	Liaoning	0.577	0.575	0.590	0.594	0.609	0.612	0.648	0.660	0.668	0.676	Marginal coordination	Preliminary coordination
	Shanghai	0.598	0.603	0.609	0.617	0.639	0.655	0.693	0.705	0.723	0.719	Marginal coordination	Intermediate coordination
	Jiangsu	0.724	0.721	0.735	0.741	0.773	0.786	0.825	0.844	0.856	0.859	Intermediate coordination	Good coordination
	Zhejiang	0.696	0.683	0.703	0.721	0.739	0.749	0.800	0.824	0.832	0.836	Preliminary coordination	Good coordination
	Fujian	0.499	0.526	0.538	0.553	0.553	0.555	0.604	0.634	0.639	0.655	Near imbalance	Preliminary coordination
	Shandong	0.718	0.719	0.744	0.726	0.755	0.783	0.813	0.843	0.861	0.895	Intermediate coordination	Good coordination
	Guangdong	0.768	0.774	0.793	0.798	0.818	0.813	0.867	0.885	0.891	0.905	Intermediate coordination	Excellent coordination
	Hainan	0.324	0.331	0.350	0.356	0.348	0.368	0.356	0.378	0.387	0.385	Mild imbalance	Mild imbalance
Central	Shanxi	0.457	0.463	0.465	0.479	0.472	0.497	0.529	0.549	0.540	0.555	Near imbalance	Marginal coordination
	Jilin	0.433	0.416	0.448	0.446	0.468	0.491	0.500	0.517	0.521	0.524	Near imbalance	Marginal coordination
	Heilongjiang	0.422	0.429	0.437	0.453	0.464	0.480	0.491	0.503	0.519	0.527	Near imbalance	Marginal coordination
	Anhui	0.508	0.529	0.538	0.542	0.549	0.575	0.611	0.626	0.637	0.648	Marginal coordination	Preliminary coordination
	Jiangxi	0.475	0.464	0.477	0.481	0.484	0.491	0.569	0.586	0.593	0.608	Near imbalance	Preliminary coordination
	Henan	0.590	0.545	0.565	0.585	0.590	0.599	0.662	0.691	0.713	0.723	Marginal coordination	Intermediate coordination
	Hubei	0.550	0.558	0.568	0.570	0.598	0.625	0.655	0.681	0.677	0.693	Marginal coordination	Preliminary coordination
	Hunan	0.506	0.518	0.525	0.540	0.554	0.568	0.649	0.671	0.672	0.687	Marginal coordination	Preliminary coordination
Western	Inner Mongolia	0.412	0.407	0.417	0.425	0.443	0.451	0.462	0.468	0.481	0.485	Near imbalance	Near imbalance
	Guangxi	0.426	0.427	0.445	0.442	0.454	0.463	0.488	0.509	0.512	0.519	Near imbalance	Marginal coordination
	Chongqing	0.481	0.479	0.508	0.487	0.520	0.529	0.541	0.573	0.580	0.585	Near imbalance	Marginal coordination
	Sichuan	0.573	0.566	0.582	0.592	0.597	0.614	0.645	0.649	0.674	0.680	Marginal coordination	Preliminary coordination
	Guizhou	0.347	0.362	0.366	0.384	0.385	0.408	0.444	0.450	0.448	0.455	Mild imbalance	Near imbalance
	Yunnan	0.371	0.371	0.388	0.384	0.396	0.401	0.416	0.430	0.435	0.459	Mild imbalance	Near imbalance
	Tibet	0.151	0.156	0.129	0.165	0.180	0.185	0.177	0.183	0.206	0.190	Severe imbalance	Severe imbalance
	Shaanxi	0.492	0.505	0.510	0.520	0.518	0.540	0.565	0.589	0.594	0.598	Near imbalance	Marginal coordination
	Gansu	0.312	0.322	0.338	0.335	0.339	0.357	0.395	0.409	0.418	0.425	Mild imbalance	Near imbalance
	Qinghai	0.204	0.198	0.201	0.226	0.227	0.236	0.251	0.259	0.250	0.259	Moderate imbalance	Moderate imbalance
	Ningxia	0.288	0.292	0.303	0.308	0.322	0.324	0.345	0.349	0.350	0.352	Mild imbalance	Mild imbalance
	Xinjiang	0.372	0.374	0.372	0.383	0.388	0.400	0.411	0.423	0.428	0.443	Mild imbalance	Near imbalance

From the regional level, the changes in coupling coordination degree across the eastern, central, and western regions demonstrated significant spatial differentiation characteristics, with average annual growth rates of 2.087, 2.305, and 2.332%, respectively. The eastern region maintained levels at or above preliminary coordination in most years; the central region achieved a phased transition from near imbalance to marginal coordination; although the western region gradually improved from a state of near imbalance, a pronounced gap remained compared to the eastern and central regions. In 2023, the coupling coordination degree levels across regions exhibited a gradient distribution pattern of “eastern > national > central > western,” and this regional disparity was closely related to factors such as policy support intensity, economic development level, and demographic structure, indicating that China’s coupling coordination level between older adult care service resource supply and demand still had considerable room for improvement.

From the provincial level, from 2014 to 2023, provinces such as Jiangsu, Zhejiang, Shandong, and Guangdong occupied leading positions nationwide, with average coupling coordination degrees reaching 0.791, 0.766, 0.806, and 0.837, respectively, all approaching or entering the high-level coupling stage. In contrast, Tibet and Ningxia had average coupling coordination degrees of only 0.170 and 0.320, respectively, still remaining in the low-level coupling stage, while most other provinces and municipalities were predominantly in the antagonistic or running-in stages. This pattern indicated that significant inter-regional disparities existed in the coupling coordination level between older adult care service resource supply and demand in China, with a limited number of provinces and municipalities achieving high coordination levels, overall exhibiting a stepped distribution characteristic of “eastern > central > western” with higher levels in the east and lower levels in the west.

**Figure 4:**
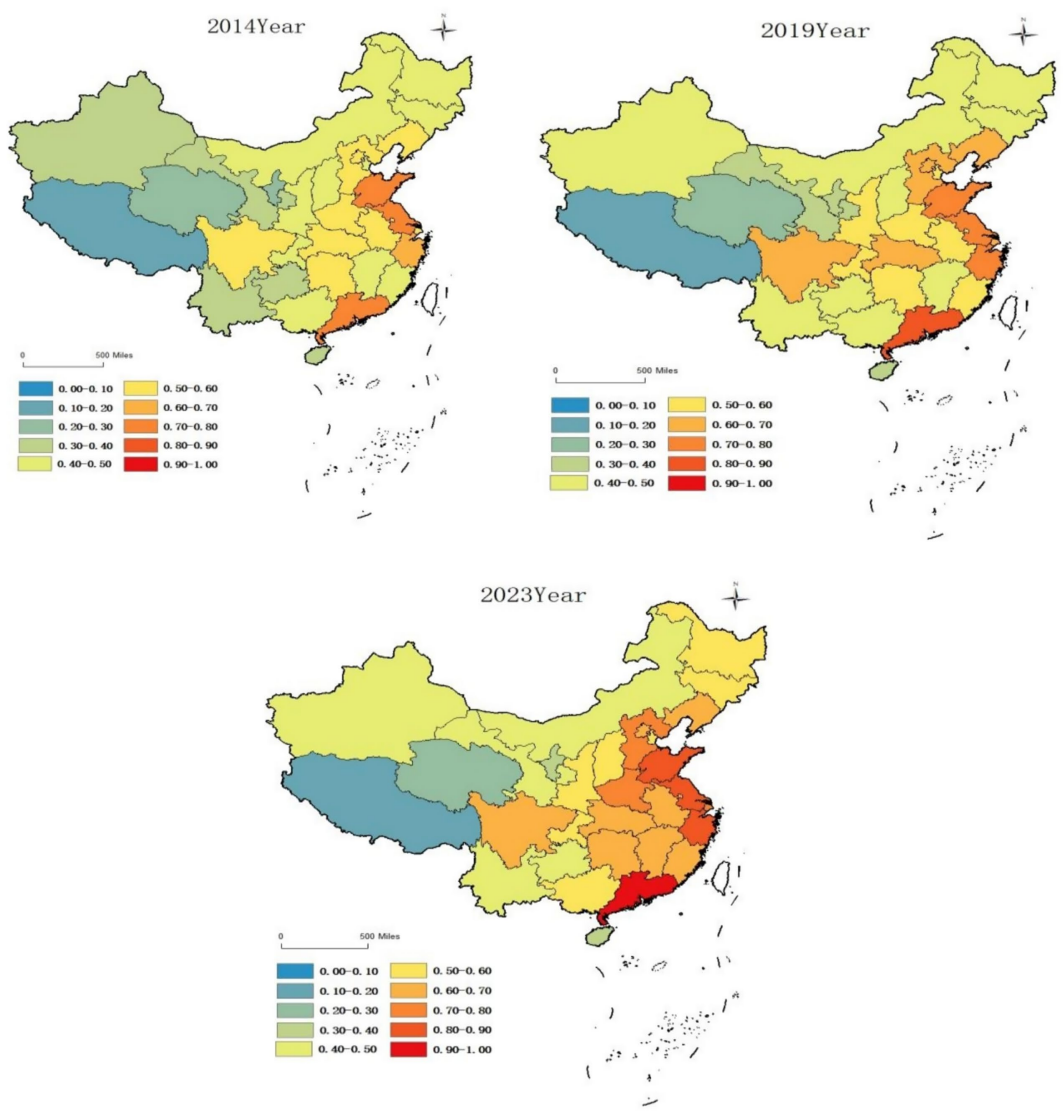
Spatial distribution map of coupling coordination degree across regions in China.

Based on the spatial distribution of coupling coordination degree levels between older adult care service resource supply and demand across provinces and municipalities in China (Figure 4), the degree of regional coordinated development from 2014 to 2023 exhibited pronounced spatial disparities, specifically manifested as a stepped distribution characteristic of higher levels in the east and lower levels in the west.

In 2014, provinces in the eastern region were generally in the marginal coordination stage, with Jiangsu, Shandong, and Guangdong having already reached intermediate coordination levels, while Hainan remained in a state of mild imbalance. Provinces in the central region were primarily distributed between the near imbalance and marginal coordination stages. Most provinces in the western region were in a state of imbalanced older adult care service resource supply and demand, specifically: Guizhou, Yunnan, Gansu, and Xinjiang were in the mild imbalance stage; Qinghai and Ningxia were in the moderate imbalance stage; and Tibet was in the severe imbalance stage. By 2018, the coordination degree of older adult care service resource supply and demand across all regions nationwide had improved, with the overall development trend continuing positively.

By 2023, China’s coupling coordination degree between older adult care service resource supply and demand had achieved significant improvements. Most provinces in the eastern region had advanced to preliminary coordination levels and above, with Guangdong having achieved excellent coordination levels. Most provinces in the central region had entered marginal coordination levels and above, with half of the provinces having reached preliminary coordination levels and above. However, the coupling coordination status of older adult care service resource supply and demand in the western region remained concerning, with most provinces still hovering at the edge of imbalance. Tibet, Qinghai, and Ningxia, in particular, remained persistently in low-level coupling development stages.

### 4.3 Dynamic evolution of coupling coordination degree between older adult care service resource supply and demand in China

**Figure 5:**
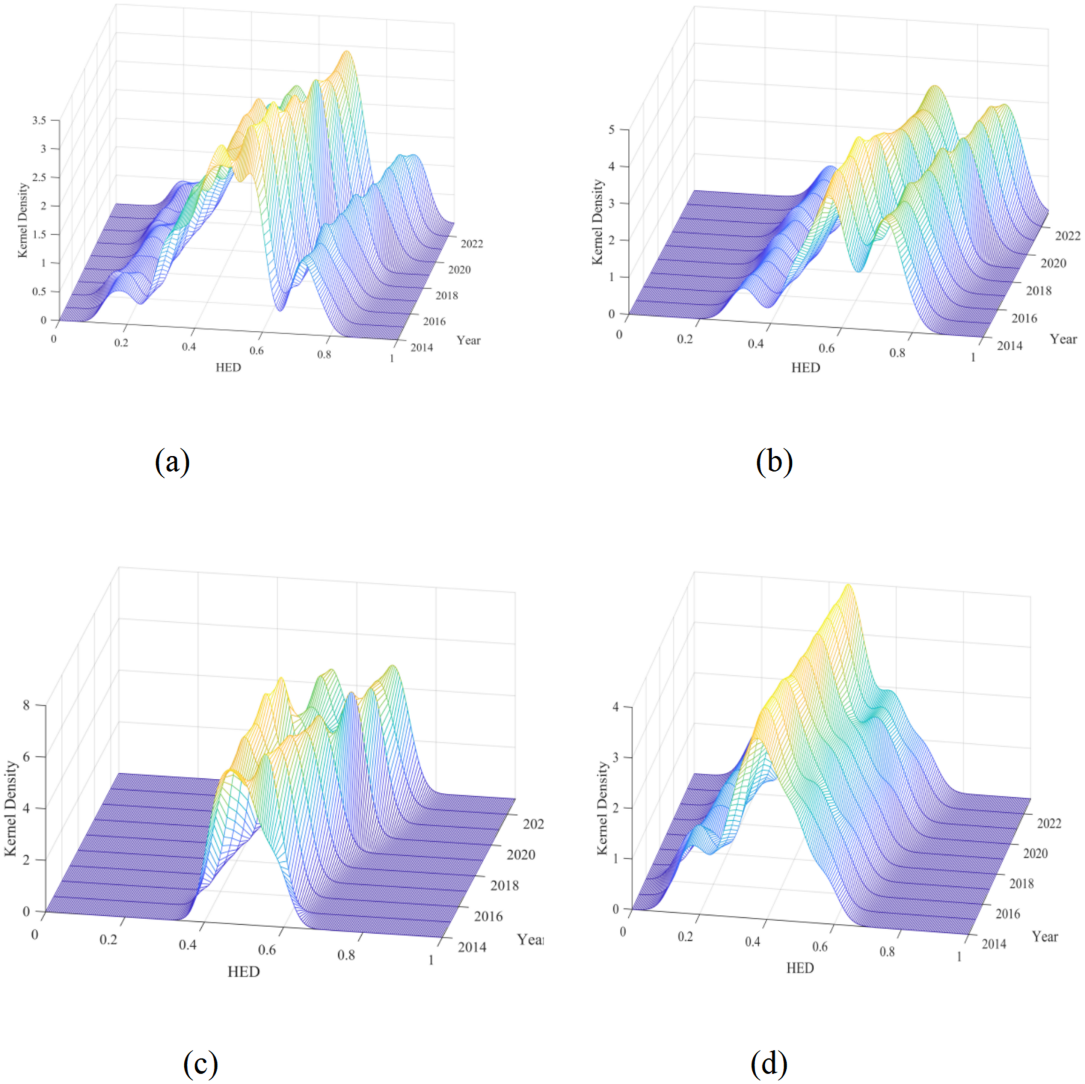
Kernel density estimation of coupling coordination degree in China from 2014 to 2023. **(a)** Nationwide coupling coordination degree; **(b)** East coupling coordination degree; **(c)** Central coupling coordination degree; **(d)** West coupling coordination degree.

The dynamic evolution of the distribution of coupling coordination degree between older adult care service resource supply and demand in China and the three major regions (Figure 5) revealed that, from the perspective of overall distribution dynamics, the center of the kernel density curve for the coupling coordination degree of older adult care service resource supply and demand nationwide shifted rightward overall, indicating an upward trend in coupling coordination levels across the country. From the perspective of distribution morphology, the curve consistently exhibited a bimodal distribution characteristic throughout the study period, indicating that polarization in coupling coordination levels persistently existed at the national level. From the perspective of distribution extensibility, the right-tail phenomenon of the curve intensified annually, indicating that high-level provinces further developed, with the gap between them and other provinces gradually widening.

From the perspective of distribution dynamics across the three major regions, the center of the kernel density curve for the eastern region continuously shifted rightward, indicating steady improvement in the coupling coordination level between older adult care service resource supply and demand in this region. The peak height of the curve increased annually, while the width first contracted and then slightly expanded. The distribution center of the curve stabilized in the 0.6–0.7 range, with overall coordination levels relatively high and development relatively stable.

The center of the kernel density curve for the central region exhibited fluctuating movement characteristics, indicating phased fluctuations in the coupling coordination level between older adult care service resource supply and demand in this region. The curve transformed from a single-peak distribution in the early study period to a bimodal distribution in the later period, with the distance between the two peaks gradually expanding, indicating that the polarization phenomenon in coupling coordination levels in the central region gradually emerged and intensified. The peak height of the curve fluctuated, reflecting increasing inter-regional disparities.

The center of the kernel density curve for the western region underwent complex changes of “rightward-leftward-rightward,” with relatively small movement amplitude, indicating that the coupling coordination level between older adult care service resource supply and demand in this region fluctuated considerably, with relatively weak developmental stability. The overall distribution center of the curve was located around 0.4, a relatively low level. The curve transformed from a single-peak distribution in the early study period to a bimodal distribution in the later period, indicating that the coupling coordination level between older adult care service resource supply and demand in the western region shifted from relative concentration toward differentiation.

In summary, during the period 2014–2023, the coupling coordination degree between older adult care service resource supply and demand at both the national and major regional levels exhibited distinct dynamic evolutionary characteristics. While overall coordination levels improved at the national level, the polarization phenomenon persisted. The eastern region maintained steady development, the polarization phenomenon in the central region gradually emerged and intensified, while the western region shifted from concentration toward differentiation with overall levels remaining relatively low, requiring further strengthening of policy support and optimization of resource allocation to promote more balanced and coordinated development.

### 4.4 Analysis of influencing factors of coupling coordination degree between older adult care service resource supply and demand in China

This section, based on a two-way fixed-effects Tobit panel model (Table 5), systematically analyzes the impacts of economic development level, government support intensity, sociodemographic structure, social capital investment, education expenditure, and older adult consumption capacity on the coupling coordination degree between older adult care service resource supply and demand. The model simultaneously controlled for provincial fixed effects and time fixed effects, effectively mitigating omitted variable bias and temporal trend interference.

**Table 5:** Tobit regression results with two-way fixed effects.

Variable	Nationwide	Eastern	Central	Western
PGD	0.2952^*^	−0.2265^*^	−0.0330^*^	0.0342
	(2.2459)	(−2.1439)	(−2.2774)	(0.4650)
GES	0.0626^*^	0.0368	−0.0005	−0.0013
	(2.2916)	(1.7565)	(−0.1552)	(−0.1214)
ODR	0.0036	−0.1171	−0.0561^**^	0.0554^**^
	(0.0877)	(−1.0534)	(−2.7191)	(2.6430)
SCR	0.1917^***^	0.1040	0.0323^*^	0.0039
	(3.6025)	(0.7184)	(2.0164)	(0.1597)
FEE	0.0065	0.4127^***^	0.0635^**^	0.0329
	(0.0617)	(4.0466)	(2.8840)	(0.6140)
SDI	0.0249	0.0459	0.0224	0.0049
	(1.6871)	(0.3217)	(1.5863)	(0.8957)
cons	−0.6293^*^	3.6616^**^	0.3333^*^	0.0994
	(−2.1441)	(2.7511)	(2.4549)	(0.5272)
$\sigma^{2}$	0.0002^***^	0.0056^***^	0.0001^***^	0.0000^***^
	(12.0414)	(7.4162)	(6.3246)	(7.7460)
N	310	110	80	120

t statistics in parentheses. *p < 0.05, **p < 0.01, ***p < 0.001.

#### 4.4.1 Economic development level

At the national level, per capita GDP (PGD) exerted a significantly positive effect on coupling coordination degree (*β* = 0.2952, *p* < 0.05), indicating that economic development served as a fundamental driver for promoting supply–demand coordination. Regional differences were significant: the eastern region exhibited a significantly negative effect (*β* = −0.2265, *p* < 0.05), the central region was similarly negative (*β* = −0.0330, *p* < 0.05), while the western region was positive but not significant (*β* = 0.0342). The negative effect in the eastern region may reflect that after economic development entered a mature stage, the marginal contribution of incremental economic growth to coordination degree diminished, or even structural imbalances emerged due to excessive supply. Although the central region was significantly negative, the absolute value of the coefficient was relatively small, indicating a transitional stage. Although not significant in the western region, the positive coefficient still reflected the potential promotional effect of economic growth.

#### 4.4.2 Government support intensity

At the national level, government fiscal expenditure on older adult care services (GES) significantly promoted coupling coordination between older adult care service resource supply and demand (*β* = 0.0626, *p* < 0.05). Examining regional variations, the coefficients for the eastern, central, and western regions were 0.0368, −0.0005, and −0.0013, respectively, all nonsignificant. This indicated that after controlling for fixed effects, the regionally heterogeneous effects of government expenditure weakened. Although positive but not significant in the eastern region, this may have been because market mechanisms were relatively well-developed, and the marginal effect of government investment was limited. The negative but nonsignificant results in the central and western regions suggested that the allocation efficiency and structure of fiscal investment required optimization.

#### 4.4.3 Sociodemographic structure

At the national level, older adult dependency ratio (ODR) had no significant effect on coupling coordination degree (*β* = 0.0036). The regional level exhibited pronounced differentiation: the eastern region was not significant (*β* = −0.1171), the central region was significantly negative (*β* = −0.0561, *p* < 0.01), and the western region was significantly positive (*β* = 0.0554, *p* < 0.01). The negative effect in the central region may have reflected a mismatch between population aging pressure and service supply capacity growth. The positive effect in the western region indicated that in regions with weak foundations, increased aging intensity could compel older adult care service system construction, promoting supply–demand coordination.

#### 4.4.4 Social capital investment

At the national level, the proportion of social capital investment in older adult care industry (SCR) significantly promoted coupling coordination (*β* = 0.1917, *p* < 0.001), representing one of the most influential factors. Examining regional variations, only the central region was significantly positive (*β* = 0.0323, *p* < 0.05), while the eastern and western regions were both nonsignificant. This indicated that social capital played an effective complementary market role in the central region, while in the eastern and western regions, its role was constrained by differences in market maturity or capital activity.

#### 4.4.5 Education expenditure investment

At the national level, fiscal education expenditure (FEE) had no significant effect on coupling coordination degree (*β* = 0.0065). However, regional differences were extremely significant: both the eastern (*β* = 0.4127, *p* < 0.001) and central (*β* = 0.0635, *p* < 0.01) regions were significantly positive, while the western region was not significant (*β* = 0.0329). The eastern region had the largest coefficient, indicating that education investment, through cultivating professional talent and enhancing service awareness, constituted an important pillar of high coordination levels in this region. Education investment in the central region likewise effectively promoted coordinated development. Educational resources in the western region were relatively scarce, and their promoting role had not yet fully manifested.

#### 4.4.6 Older adult consumption capacity

At both the national and regional levels, per capita disposable income of older adult (SDI) did not exhibit significant effects. The national coefficient was 0.0249 (*p* > 0.1), with the eastern, central, and western regions at 0.0459, 0.0224, and 0.0049, respectively, all nonsignificant. This suggested that after controlling for fixed effects, the direct pulling effect of consumption capacity weakened, possibly because it operated indirectly through other variables or because supply-side constraints limited demand conversion.

#### 4.4.7 Robustness testing

A two-way fixed-effects Tobit model with one-period lagged explanatory variables was employed for robustness testing (detailed results provided in Supplementary materials). Results showed that the coefficient directions and significance of core variables remained largely stable: at the national level, PGD (*β* = 0.3072, *p* < 0.05) and GES (*β* = 0.0580, *p* < 0.05) remained significantly positive. At the regional level, education expenditure maintained significantly positive effects in the eastern (*β* = 0.0453, *p* < 0.001) and central (*β* = 0.0551, *p* < 0.05) regions. Although sociodemographic structure in the western region was no longer significant, the direction remained consistent. The σ^2^ values for both models were relatively small and highly significant, indicating high model estimation precision. The consistency between the lagged one-period results and the main model verified the robustness of this study’s conclusions.

Comprehensive analysis revealed that social capital investment and education expenditure constituted key factors promoting coupling coordination, with effects exhibiting significant regional heterogeneity. Economic development level and government support played positive roles at the national level, but regional effects were complex. Overall model validity was verified, providing empirical evidence for differentiated policy formulation.

## 5 Discussion and recommendations

### 5.1 Discussion

Based on panel data from 31 provinces and municipalities in China from 2014 to 2023, this study constructed a systematic analytical framework for evaluating the coupling coordination of older adult care service resource supply and demand. The study employed the entropy weight method for objective weighting to construct a comprehensive evaluation indicator system for supply and demand, utilized the relative development degree model to identify the coordination status of supply–demand development, measured the coordinated development level of the two systems through the coupling coordination degree model, employed kernel density estimation to depict the spatiotemporal distribution and dynamic evolution characteristics of coupling coordination degree, and applied a Tobit panel model with provincial and time two-way fixed effects to analyze in depth the key driving factors influencing coupling coordination development, while conducting robustness testing using a Tobit model with one-period lagged explanatory variables. The study yielded the following main conclusions:

#### 5.1.1 Overall coupling coordination level still has significant room for improvement

From 2014 to 2023, although the coupling coordination degree between older adult care service resource supply and demand in China exhibited an upward trend, it remained overall in the range from near imbalance to marginal coordination. This phenomenon stemmed from the relatively late start of China’s older adult care service system construction, with the long-established family care tradition resulting in lagging development of the socialized older adult care service supply system (42). Rapid economic development following reform and opening-up provided a material foundation for older adult care service resource accumulation, but supply-side institutional construction, resource allocation, and service model innovation did not keep pace. On the demand side, the accelerated population aging process overlapped with family structural changes brought about by the family planning policy. The traditional “4-2-1” family structure weakened family care functions, yet older adults care concepts transformed relatively slowly, with their awareness and acceptance of socialized older adult care services still requiring time for cultivation (43). Regional disparities significantly reflected the continuation of China’s unbalanced development. The eastern region maintained marginal coordination primarily benefiting from the economic foundation and public service system accumulation resulting from the priority development strategy since reform and opening-up, while the central and western regions’ persistent state of imbalance reflected the influence of evolving regional development strategies. Improving overall coordination levels requires not only increased resource investment but also institutional-level improvements to older adult care service supply–demand coordination mechanisms and promotion of care concept transformation and service model innovation.

#### 5.1.2 Provincial level exhibits significant differentiation, with breakthroughs achieved in some regions

The significant differentiation at the provincial level reflected the long-term accumulation effects of regional development strategies since reform and opening-up. Provinces such as Jiangsu, Zhejiang, Shandong, and Guangdong had already entered preliminary coordination or even good coordination stages. These provinces completed industrialization and urbanization processes relatively early, accumulating substantial economic foundations and comprehensive public service systems. Regional integration policies further strengthened resource agglomeration effects, promoting the formation of older adult care service industry clusters and digital technology application (44). In contrast, the western region’s overall lag stemmed from the superposition of multiple factors. Geographical environmental constraints limited population agglomeration and industrial development, weak transportation infrastructure increased service supply costs, and prolonged positioning in a relatively marginal role in national development strategies resulted in obvious shortcomings in infrastructure and talent reserves. Although the Western Development Strategy had been implemented for many years, the intensity of targeted support in the older adult care service field remained relatively limited. Cultural factors likewise could not be ignored. Family care concepts in western minority regions were more traditional, with lower acceptance of socialized older adult care services, and language and cultural differences also increased service supply difficulty. The central region was in a transitional state, with its east–west connecting geographical advantage not yet fully transformed into older adult care service development advantages, and the problem of aging before becoming affluent caused by sustained labor outflow further intensified supply–demand contradictions.

#### 5.1.3 Economic structural transformation and fiscal system constraints on regional coordinated development

The regional heterogeneity revealed by the two-way fixed-effects Tobit model profoundly reflected the staged differences in economic structural transformation and structural contradictions in the fiscal system. The impact of economic development level on coordination degree exhibited entirely different characteristics across the three major regions. The significantly negative effect in the eastern region stemmed from its entry into the post-industrial stage, with economic growth mode shifting toward innovation-driven development, yet older adult care service supply continuing the scale expansion model, causing structural imbalances between high-end facility vacancies and grassroots service insufficiency. The central region likewise exhibited a negative effect but with a smaller coefficient, reflecting its transitional stage of industrial structural adjustment, with the driving effect of economic growth on older adult care service improvement weakening. Although the western region exhibited a positive effect, it was not significant, rooted in its position in the early stage of industrialization, with older adult care service system construction relatively lagging and economic growth not yet effectively transformed into service supply capacity. Fiscal support significantly promoted coordination at the national level, but regional effects were all nonsignificant, revealing resource allocation efficiency issues under the tax-sharing fiscal system. The eastern region had high fiscal self-sufficiency but relatively low older adult care service investment proportions with diminishing marginal effects. Although the central and western regions were main beneficiaries of transfer payments, constrained by insufficient local fiscal matching capacity and expenditure rigidity, special fund utilization efficiency was suboptimal. Local government dependence on land finance following tax-sharing reform further crowded out public service sector investment. The differentiation effect of sociodemographic structure reflected the influence of population mobility patterns. The significantly negative effect of older adult dependency ratio in the central region reflected the predicament of aging before becoming affluent caused by labor outflow, with young and middle-aged individuals migrating to the east, increasing left-behind older adult populations while service supply capacity construction lagged. The significantly positive effect in the western region indicated that under relatively small population bases, increased aging intensity compelled greater government investment, forming a demand-driven supply mechanism (45).

#### 5.1.4 Market mechanisms and educational resource distribution influence factor allocation efficiency

The regional differences in social capital investment and education expenditure essentially reflected imbalances in market mechanism maturity and higher education resource distribution. Social capital investment had the strongest influence at the national level but was significant only in the central region. This pattern stemmed from differences in marketization levels across regions. The older adult care service market in the eastern region had already matured, with limited space for social capital entry and facing relatively high competitive pressures. The central region was in the deepening stage of market-oriented reform, with coexistence of government dominance and market mechanisms, allowing social capital to effectively fill public service supply gaps. In the western region, market mechanisms were underdeveloped, with low social capital activity, and combined with long investment return cycles and high risks, local governments’ lack of supporting policies further constrained social capital participation. The extremely significant regional differences in education expenditure profoundly reflected the historical pattern of key universities and research institutions being primarily located in eastern metropolises since the founding of the nation. The eastern region relied on abundant higher education resources to establish comprehensive older adult care service talent cultivation systems, with close industry-university-research cooperation (46). Although the central region had some key universities, overall quantity and quality fell short of the east. The western region lacked higher education resources, with insufficient talent cultivation capacity and facing serious talent outflow problems, with excellent graduates tending to seek employment in developed eastern regions, further weakening the local effects of education investment. The nonsignificance of older adult consumption capacity at both national and regional levels reflected the universal existence of supply-side constraints, meaning that even when older adults possessed payment capacity, demand could not effectively convert without high-quality service supply.

### 5.2 Recommendations

#### 5.2.1 Construct regionally differentiated fiscal support systems to improve resource allocation efficiency

We recommend establishing a centrally coordinated, classified fiscal support system. The western region should establish special transfer payments for older adult care services, adopting factor-based methods for fund allocation, with priority given to provinces with large older adult populations and low fiscal self-sufficiency, supporting digital transformation of older adult care institutions, construction of community day care centers, and development of integrated medical-nursing services (47). The central region should optimize existing fiscal expenditure structures, establishing mechanisms linking performance evaluation with fund allocation, focusing on supporting older adult care service information platform construction and professional talent cultivation. The eastern region should adjust fiscal investment directions, shifting from scale expansion to quality enhancement, concentrating resources on supporting home-based and community-based older adult care service development while avoiding excessive construction of high-end institutions. Simultaneously, provincial-level coordination mechanisms should be improved, older adult care service development funds established, and social capital participation attracted through PPP models and service purchasing, forming synergistic effects between fiscal funds and social capital.

#### 5.2.2 Cultivate social capital participation mechanisms by category to stimulate market entity vitality

We recommend implementing differentiated cultivation strategies to stimulate social capital vitality (48). The central region should consolidate and expand social capital participation advantages, establishing standardized market entry and exit mechanisms, improving service standards and quality supervision systems, and reducing operational costs through preferential tax policies, land security, and financing support to encourage development of chain and brand older adult care service enterprises. The western region should focus on improving the business environment, reducing investment risks through government service purchasing, risk compensation, and loan interest subsidies, prioritizing support for basic and inclusive older adult care service projects, and exploring east–west older adult care service enterprise partnership models. The eastern region should guide social capital to focus on service innovation and quality improvement, supporting development of emerging formats such as smart older adult care and wellness tourism. A unified national older adult care service market credit system should be established, social capital entry and exit mechanisms improved, and older adult rights and service quality protected.

#### 5.2.3 Promote balanced allocation of educational resources and establish talent mobility incentive mechanisms

We recommend implementing balanced educational resource allocation initiatives to address talent shortcomings. At the central level, support should be provided for key universities in central and western regions to develop disciplines such as gerontology, rehabilitation medicine, and health management, with increased enrollment plans and funding investment, and educational quality improved through counterpart support and faculty training (49). At the local level, vocational education investment should be increased, with older adult care service vocational colleges established in prefecture-level cities to cultivate skilled talent. Regional talent mobility incentive mechanisms should be established, providing preferential policies such as salary subsidies, housing support, and children’s education benefits for professional talent working in older adult care services in central and western regions. Industry-education integration should be promoted, supporting older adult care service enterprises and universities to jointly establish training bases and conduct order-based talent cultivation. Continuing education systems for older adult care service practitioners should be established, professional title evaluation and career development pathways improved, and the attractiveness of older adult care service professions enhanced.

#### 5.2.4 Optimize population spatial distribution policies to promote rational allocation of older adult care service resources

We recommend optimizing population spatial distribution policies to address regional differentiation challenges. The central region should attract population return migration through developing distinctive industries and improving employment environments, while accelerating construction of home-based and community-based older adult care service networks, enhancing grassroots service supply capacity, and promoting in-depth implementation of integrated medical-nursing care at county and township levels. The western region should leverage the positive effects of aging-driven mechanisms, increasing older adult care service infrastructure investment, promoting equalization of urban–rural basic older adult care services, focusing on addressing rural older adult care service gaps, and exploring locally appropriate service models such as mutual-aid older adult care and volunteer services (50). The eastern region should optimize urban spatial layout, developing polycentric networked urban structures, alleviating older adult care service pressure in central urban areas, and promoting downward extension of older adult care services to communities. East–west older adult care service partnership mechanisms should be established, cross-regional older adult care service models explored, cross-regional flow and optimal allocation of older adult care service resources promoted, and population mobility and pension insurance portability systems improved.

## 6 Research limitations and future prospects

Although this study conducted a systematic analysis of the coupling coordination development of older adult care service resource supply and demand in China, several limitations remain, pointing toward directions for future research. First, this study did not fully examine spatial dependence and spatial spillover effects. The allocation of older adult care service resources and policy diffusion possess significant spatial correlation characteristics, with mutual influences among adjacent provinces in development models, policy innovation, and resource flows. Although the two-way fixed-effects Tobit model controlled for provincial fixed effects and time fixed effects, it did not explicitly test spatial interaction mechanisms. Future research will introduce spatial econometric models (such as spatial Durbin models and spatial panel Tobit models) to systematically test spatial dependence, identify direct effects and spillover effects between regions, and analyze in depth the spatial transmission mechanisms of older adult care service resource supply–demand coordination. Second, the evaluation indicator system was constrained by data availability, with some key indicators lacking systematic data at the provincial level. Future research will integrate micro-survey data to construct a more comprehensive evaluation system. Third, the influencing factor analysis primarily focused on the macro level, with relatively limited exploration of deeper factors such as sociocultural influences and policy interaction effects. Future research will introduce multilevel data to explore in depth micro-entity behavioral decision-making mechanisms. Additionally, this study will form the basis for ongoing research, continuously tracking the dynamic evolution of China’s older adult care service system development to provide sustained theoretical support and empirical evidence for constructing precise and effective policy intervention systems.

## Data Availability

The raw data supporting the conclusions of this article will be made available by the authors, without undue reservation.
