# Supplementary material for: Coupling coordination evaluation and influencing factors analysis of older adult care service resource supply and demand in China
Source: Front Public Health. 2025 Nov 26;13:1662064. doi: 10.3389/fpubh.2025.1662064 (PMC12690935; doi:10.3389/fpubh.2025.1662064)
Supplement: Supplementary file 1 [file Data_Sheet_1.pdf]

Supplementary Materials

I. Robustness Testing Analysis of Lagged One-Period Tobit Model

1. Model Specification and Testing Purpose

To verify the robustness of the main model results and further mitigate potential endogeneity issues, this study employed a two-way fixed-effects Tobit model with one-period lagged explanatory variables for robustness testing. By introducing lagged terms of explanatory variables, this model could to some extent reduce endogeneity bias caused by contemporaneous bidirectional causality while retaining provincial and time fixed effects to control for time-invariant provincial characteristics and temporal trends. The lagged one-period model sample size comprised 279 observations (nationwide), 99 observations (eastern region), 72 observations (central region), and 108 observations (western region). Compared to the main model, the reduction of 31 observations resulted from missing first-year observations due to lagging procedures.

2. Core Variable Comparison Analysis

The regression results of the lagged one-period model generally supported the core findings of the main model but exhibited certain differences in statistical significance and effect magnitude for some variables. Specifically, economic development level (PGD) maintained a significantly positive effect at the national level (β=0.3072, $p<0.05$), with coefficient direction completely consistent with the main model. However, the negative effects in the eastern and central regions were no longer significant in the lagged one-period model, which might reflect that the impact of economic development on older adult care service coordination exhibited certain lag effects, with current economic conditions potentially absorbed by other factors in the subsequent period. Government fiscal expenditure (GES) likewise maintained a significantly positive effect at the national level (β=0.0580, $p<0.05$), and exhibited a significantly positive effect in the eastern region (β=0.0046, $p<0.05$), which differed slightly from the nonsignificant result in the eastern region in the main model, indicating that the positive effect of fiscal expenditure in the eastern region might possess lagged characteristics.

Sociodemographic structure (ODR) was nonsignificant at both the national and regional levels in the lagged one-period model, differing from the significantly negative result in the central region and significantly positive result in the western region in the main model. This suggested that the impact of demographic structure variables on coupling coordination might primarily manifest as contemporaneous effects. Social capital investment (SCR) was nonsignificant at both the national and regional levels in the lagged one-period model, presenting a notable difference from the main model results. This might indicate that the positive effect of social capital investment on coupling coordination primarily operated through contemporaneous mechanisms, with relatively limited lagged effects. Education expenditure (FEE) maintained significantly positive effects in the eastern region (β=0.0453, $p<0.001$) and central region (β=0.0551, $p<0.05$), with coefficient directions completely consistent with the main model. However, it should be noted that the eastern region coefficient decreased substantially from 0.4127 in the main model to 0.0453, a decline of approximately 89%. The central region coefficient decreased modestly from 0.0635

to 0.0551, with significance level declining from p<0.01 to p<0.05. These changes might reflect that the impact of education investment on coupling coordination exhibited strong immediate effects, while lagged effects, although still significant, were substantially weakened in magnitude. Older adult consumption capacity (SDI) exhibited a significantly positive effect in the western region (β=0.0125, $p<0.05$), differing from the nonsignificant results at both national and regional levels in the main model, potentially reflecting that income enhancement exhibited certain lag effects on older adult care service demand conversion in the western region.

3. Robustness Testing Conclusions

Overall, the estimation results of the lagged one-period two-way fixed-effects Tobit model remained largely consistent with the main model in terms of coefficient directions for core variables, with the positive effects of key variables such as economic development level, government fiscal expenditure, and education expenditure being further validated in the lagged one-period model. Changes in statistical significance for some variables primarily reflected differences in the temporal lag characteristics of different influencing factors, which did not undermine the robustness of the main model's core conclusions. The model's σ^2 values were relatively small and highly significant, indicating high model estimation precision. Overall, the robustness testing results of the lagged one-period model supported the main findings of this study, validating the regionally heterogeneous impacts of economic structural transformation, fiscal system constraints, market mechanism development, and educational resource distribution on the coupling coordination development of older adult care service resource supply and demand, providing a more reliable empirical foundation for policy recommendations.

Table 5. (Supplementary Materials): Lagged Tobit Regression Results with Two-way Fixed Effects

Variable	Nationwide	Eastern	Central	Western
PGD	0.3072*	-0.0197	-0.0130	0.0659
	(2.3693)	(-1.8596)	(-0.8472)	(0.8847)
GES	0.0580*	0.0046*	0.0025	-0.0174
	(2.2205)	(2.1908)	(0.7326)	(-1.6802)
ODR	0.0502	0.0071	-0.0376	0.0029
	(1.2338)	(0.6396)	(-1.6460)	(0.1372)
SCR	0.0911	0.0072	-0.0187	0.0017
	(1.7897)	(0.5094)	(-1.1382)	(0.0683)
FEE	0.0571	0.0453***	0.0551*	-0.0827
	(0.5401)	(4.2696)	(2.3946)	(-1.4346)
SDI	0.0241	0.0146	-0.0010	0.0125*
	(1.6665)	(1.0264)	(-0.0660)	(2.1864)
cons	-0.6534*	0.2477	0.2900	0.3233
	(-2.2730)	(1.8710)	(1.9813)	(1.7004)
σ^2	0.0002***	0.0001***	0.0001***	0.0000***
	(11.4232)	(7.0356)	(6.0000)	(7.3485)
N	279	99	72	108

Note: t statistics in parentheses. * $p < 0.05$, ** $p < 0.01$, *** $p < 0.001$
